# Supplementary material for: When, why and how foot orthoses (FOs) should be prescribed for children with flexible pes planus: a Delphi survey of podiatrists
Source: PeerJ. 2018 Apr 16;6:e4667. doi: 10.7717/peerj.4667 (PMC5907774; doi:10.7717/peerj.4667)
Supplement: Data S1 [file peerj-06-4667-s001.pdf]

University of South Australia

Additional file 2: Information sheet and invitation email sent to participants.

INVITATION E-MAIL

A Delphi consensus of Podiatrists for prescription of Foot Orthoses (FOs) for children with flexible pes planus

Are you fascinated by the science and practice of assessing and managing pes planus (flat feet) in clinical practice and want to learn more?

Are you intrigued by prescription of foot orthoses (FOs) in children, especially regarding when, why and how foot orthoses should be prescribed in this population?

Are you an expert podiatrist with interest in prescription of foot orthoses (FOs) in children with flexible pes planus?

Are you interested to engage with and learn from experts in the field of prescription of foot orthoses in children with flexible pes planus?

If you answered yes to these questions, then we need you!

We are currently undertaking a project which aims to derive a consensus among expert podiatrists on the prescription of foot orthoses (FOs) in children with flexible pes planus and to develop consensus-based statements to direct practitioners on when, why and how foot orthoses (FOs) should be prescribed in this population. This project uses a Delphi survey approach which requires you to complete an electronic survey (round one) within four weeks of receiving the survey and then, in subsequent rounds, consider and rank your agreement with a collection of statements that are formulated from the group responses from round one. Original statements and ranking will remain confidential. Each round will need to be returned within four weeks from the original dissemination.

Who is eligible to participate?

For the purpose of this study, an ‘expert’ will be defined as a registered podiatrist with at least 10 or more years of clinical experience and currently has (at least) one of the following:

1. holds an academic position teaching paediatric theory or clinical practice within an Australian/New Zealand/United Kingdom podiatry program;
2. holds a clinical position where the practice is focused on paediatric assessment and intervention;
3. has published research on paediatric theory or FOs within the past five years.

You have been randomly selected from identified individuals that fit these criteria. Participation in this project is entirely voluntary and will be at no-cost to you. The researchers will take every care to remove identifying material as early as possible. Likewise, individuals' responses will be kept confidential by the researchers and not be identified in the reporting of the research. However, the researchers cannot guarantee the confidentiality or anonymity of material transferred by email or the internet.

Who should I contact if I have any question?

If you have any questions about this initiative, you are welcome to contact:
Dr. Helen Banwell
C8-41, City East Campus University of South Australia, Adelaide, SA 5000
Ph: +61 8 8302 1256; Fax: +61 8 8302 2766
Email: helen.banwell@unisa.edu.au

This project has been approved by the University of South Australia’s human Research Ethics Committee. If you have any ethical concerns about the project or questions about your rights as a participant please contact the Executive Officer of this Committee, tel: +61 8 8302 3118; email: vicki.allen@unisa.edu.au

PARTICIPANT INFORMATION SHEET

Project title - A Delphi consensus of Podiatrists for prescription of Foot Orthoses (FOs) for children with flexible pes planus.

Chief Investigators – Sindhrani Dars, Helen Banwell, Hayley Uden and Saravana Kumar

Dear Participant,
You are invited to participate in a research project being conducted by Ms Sindhrani Dars, from the University of South Australia. This information sheet provides an overview of the research project. Please read this carefully to make sure that you understand the content before agreeing to participate.

Background
The focus of this study will be on flexible pes planus which is characterised by lowering or absence of the medial longitudinal arch of the foot, bringing the sole of foot into partial/complete ground contact in weight bearing with a normal arch appearance in non-weight bearing (Harris et al. 2004; Roth et al. 2013). It often occurs in conjunction with hind foot eversion (Evans 2008).

What is the purpose of this research project?
There is no clear consensus in the literature on the definition, diagnosis and management of pes planus for a paediatric population (Pfeiffer et al. 2005; Roth et al. 2013; Tudor et al. 2009). Whilst opinion on how to manage paediatric flatfoot does exist with some guidance from Harris et al. 2004 and the Paediatric flat foot proforma (p-FFP) by Evans in 2008, the efficacy of different treatments is controversial and debatable (Rome et al. 2010; Pfeiffer et al. 2005). FOs are frequently used but have limited evidence to suggest their effectiveness; the evidence is most regularly based on non-symptomatic participants which may not reflect clinical practice (Rome et al. 2010). Furthermore, no guidelines exist on how orthoses should be prescribed for this population and specifically around what age. Thus the aim of this study is to build consensus-based statements on when, why and how foot orthoses (FOs) should be prescribed for paediatric pes planus to direct both future research and clinical practice.

What are the aims of the project?
The aim of this research is to derive a consensus among expert podiatrists on the prescription of foot orthoses (FOs) in children with flexible pes planus and to develop consensus-based statements to direct practitioners on when, why and how foot orthoses (FOs) should be prescribed in this population.

If I agree to participate, what will I be required to do?
The Delphi survey methodology is a structured, rigorous and established process which is commonly used to garner consensus among a group of experts in a particular field (Sinha et al. 2011; Powell 2003). It is of benefit when there is contradicting or lack of evidence on a certain topic (Vernon 2009; Van der Linde et al. 2005). Delphi surveys address confidentiality issues by maintaining anonymity of participants (Van der Linde et al. 2005).

A Delphi survey comprising of three rounds will be undertaken as part of this research. The data from each round will be analysed and collated into statements as a group response and presented back to participants for evaluation in the subsequent rounds. These following rounds will then seek agreement to the statements compiled from responses of previous rounds.
Round One: The first round will be lengthier and more time consuming than the subsequent rounds as this round is designed to gather as much information as possible from the participants on the

prescription of FOs for paediatric flexible pes planus. You will be required to complete an electronic survey (round one) within four weeks of receiving the survey

Round Two & Three: The following rounds will require less time as they will be focused on gathering agreement. You will need to consider and rank your agreement with a collection of statements that have been formulated from round one.

Original statements and ranking will remain confidential with each round needing to be returned within four weeks from the original dissemination. As this Delphi survey aims to build consensus over three rounds, your participation in all three rounds of the survey is most desirable.

When will the Delphi survey occur?
September 2016 – February 2017

The initial round (round one) has the largest time commitment for participation. It is expected that the initial round will take approximately 15 to 30 minutes to complete. Further rounds will not be as time-consuming. There will be approximately four weeks between rounds. It is expected that the survey will conclude early 2017.

What are the benefits associated with participation?
Currently there is a gap in knowledge regarding the definition, diagnosis and management of pes planus for a paediatric population, which then influences clinical practice. Participation in this research will help to address these knowledge and practice gaps. By doing so, best practice statements for children with pes planus can be provided.

What will happen to the information I provide?
Data collected from the Delphi survey will be analysed for consensus and statements of recommendations developed. All information obtained from this research will remain strictly confidential and no information which could lead to identification of any individual will be released, unless required by law. Only the investigators will have access to the data. Electronic data will be stored on a password-protected server while paper-based copies of completed questionnaires will be kept in a locked cabinet for a period of five years in a locked filing cabinet at the city east campus of the University of South Australia as required by university policy for 'general research not involving a government department'.

The researcher will take every care to remove any identifying material from responses as promptly as possible. Likewise, individuals' responses will be kept confidential by the researcher and not be identified in the reporting of the research. However, the researcher cannot formally guarantee the confidentiality or anonymity of material transferred by email or the internet.

What are my rights as a participant?
Before the commencement of this research, you will be asked to provide your consent to participate in this research. By agreeing to participate and thereby providing consent, your participation in this research will commence. Your participation is entirely voluntary and you can withdraw from this initiative at any time. Your decision to not take part or withdraw from this research will have no impact on your current position. However, please note that once you submit your survey, we are unable to remove your response as it will be impossible to identify your individual data given the anonymous nature of the survey.
As a participant, you also have the right to ask any questions about the research at any time. All participants will be provided with the final copy of collated statements and rankings in a summary report.

The research will be conducted according to the NHMRC National Statement on Ethical Conduct in Human Research, 2007.

Who should I contact if I have any question?

If you have any questions about this evaluation, you are welcome to contact:

Dr. Helen Banwell
C8-41, City East Campus University of South Australia, Adelaide, SA 5000
Ph: +61 8 8302 1256; Fax: +61 8 8302 2766
Email: helen.banwell@unisa.edu.au

This project has been approved by the University of South Australia's Human Research Ethics Committee. If you have any ethical concerns about the project or questions about your rights as a participant please contact the Executive Officer of this Committee, Tel: +61 8 8302 3118; Email: vicki.allen@unisa.edu.au

References

Evans, AM 2008, 'The flat-footed child—to treat or not to treat: what is the clinician to do?', *Journal of the American Podiatric Medical Association*, vol. 98, no. 5, pp. 386-393.

Harris, EJ, Vanore, JV, Thomas, JL, Kravitz, SR, Mendelson, SA, Mendicino, RW, Silvani, SH & Gassen, SC 2004, 'Diagnosis and treatment of pediatric flatfoot', *The Journal of Foot and Ankle Surgery*, vol. 43, no. 6, pp. 341-373.

Pfeiffer, M, Kotz, R, Ledl, T, Hauser, G & Sluga, M 2005, 'Prevalence of flat foot in preschool-aged children', *Pediatrics*, vol. 118, no. 2, Aug, pp. 634-639.

Powell, M, Seid, M & Szer, IS 2005, 'Efficacy of custom foot orthotics in improving pain and functional status in children with juvenile idiopathic arthritis: a randomized trial', *The Journal of rheumatology*, vol. 32, no. 5, pp. 943-950.

Rome, K, Ashford, RL & Evans, A 2010, 'Non-surgical interventions for paediatric pes planus', *Cochrane Database of Systematic Reviews*, no. 7, p. CD006311.

Roth, S, Roth, A, Jotanovic, Z & Madarevic, T 2013, 'Navicular index for differentiation of flatfoot from normal foot', *International orthopaedics*, vol. 37, no. 6, pp. 1107-1112.

Sinha, IP, Smyth, RL & Williamson, PR 2011, 'Using the Delphi technique to determine which outcomes to measure in clinical trials: recommendations for the future based on a systematic review of existing studies', *PLoS - Public Library of Science (Med),* vol. 8, no. 1, p. e1000393.

Tudor, A, Ruzic, L, Sestan, B, Sirola, L & Prpić, T 2009, 'Flat-footedness is not a disadvantage for athletic performance in children aged 11 to 15 years', *Pediatrics*, vol. 123, no. 3, pp. e386-e392.

Van der Linde, H, Hofstad, CJ, van Limbeek, J, Postema, K & Geertzen, JH 2005, 'Use of the Delphi Technique for developing national clinical guidelines for prescription of lower-limb prostheses', *Journal of rehabilitation research and development*, vol. 42, no. 5, pp. 693-704.

Vernon, W 2009, 'The Delphi technique: A review', *International Journal of Therapy & Rehabilitation,* vol. 16, no. 2, pp. 69-76.
