# Supplementary material for: When, why and how foot orthoses (FOs) should be prescribed for children with flexible pes planus: a Delphi survey of podiatrists
Source: PeerJ. 2018 Apr 16;6:e4667. doi: 10.7717/peerj.4667 (PMC5907774; doi:10.7717/peerj.4667)
Supplement: Data S2 [file peerj-06-4667-s002.pdf]

Additional file 3: Round One of Delphi survey

Paediatric Pes Planus - Delphi round 1

Introduction

Thank you for participating in the Delphi survey for consensus on the prescription of foot orthoses (FOs) for flexible pes planus in children. Please note that this is Round 1 of the Delphi survey and is the heaviest section where we gather as much information as possible on the use of FOs for paediatric pes planus. This first round aims to gather information and determine if consensus exists within the profession on how we assess, classify and manage flexible pes planus in children. The written responses from this round will be summarised and collated into statements. These statements will be returned to you in the subsequent round and you will be asked to consider each statement and rank your agreement (or non-agreement) with it.

Please note that you can stop the survey at any time and come back to it later as long as you are on the same computer and same log on session. However, be aware that the page you are working on will be 'blanked' therefore it is best to pause (if you wish to) at the beginning of a new page.

Please contact Sindhrani Dars at darsy009@mymail.unisa.edu.au (Mobile: 0414 710 226) or Dr Helen Banwell at helen.banwell@unisa.edu.au (Mobile: 0417 822 997) for any queries or concerns.

* 1. Have you already completed a short 'Participant's characteristics survey' sent to you via email and agreed to provide consent to participate in this research?

◯ Yes

◯ No

Paediatric Pes Planus - Delphi round 1

Unable to continue

The previous response indicates that 'Participants characteristics survey' has not been completed, meaning consent to participate has not been provided by you. Unfortunately ethical and safety considerations do not permit participation in the survey before a consent is obtained. Please notify Sindhrani Dars at <darsy009@mymail.unisa.edu.au> or (Mobile: 0414710226) and the survey with consent option will be sent to you as soon as possible.
Apologies for the inconvenience and we hope you will return soon to complete this survey.

Paediatric Pes Planus - Delphi round 1

Survey Overview

There are four sections to this survey. The first section is aimed at determining how you establish the presence of flexible *pes planus* in children during the course of your normal practice. The second section aims to determine when intervention is necessary for children with flexible *pes planus* and the final two sections aim to establish why foot orthoses may be useful for this condition and how they are prescribed.

This first section was developed based on the responses from the preliminary survey where each panellist indicated how they assess foot posture and foot function in the paediatric population. We now ask you to revisit these questions to indicate how you assess foot posture and function specific to the paediatric flexible *pes planus* population.

Please note that the focus of this study is on flexible flat feet in otherwise healthy children i.e. not associated with neurological, muscular or structural disease or abnormalities.

Please begin Section 1 by clicking on the next button below.

Section 1: Establishing the presence of flexible pes planus

1. Please indicate which of the following assessment outcomes, if any, you routinely use to determine the presence of a flexible pes planus foot posture? (More than one answer can be selected)

- [ ] Visual or measured assessment of static foot posture
- [ ] Foot posture tools (e.g. Foot posture index (FPI), Paediatric flat foot proforma (pFFF))
- [ ] Foot print indices (e.g. arch height index, Staheli's arch index)
- [ ] Diagnostic imaging (e.g. x-rays, CT scans, MRI)

Other (please specify)

2. Which static foot posture measures, if any, do you routinely use to determine the presence of a flexible pes planus foot posture in children? (More than one answer can be selected)

- [ ] I do not determine static foot posture
- [ ] Rearfoot position (RCSP & NCSP)
- [ ] Forefoot to rearfoot relationship
- [ ] Navicular height (truncated to foot length)
- [ ] Navicular height (non-truncated)
- [ ] Navicular drift
- [ ] Navicular drop

Other (please specify)

3. When conducted, your measure of the Static Foot Posture is by? (More than one answer can be selected)

- [ ] I do not determine static foot posture
- [ ] Eyeballing
- [ ] Tractograph
- [ ] Gravity goniometer

Other (please specify)

4. Please indicate which of the following techniques, if any, you would routinely use to determine foot function in paediatric flexible pes planus populations? (More than one answer can be selected)

- [ ] I do not determine foot function
- [ ] Visual gait analysis
- [ ] Joint axes evaluation
- [ ] Supination resistance
- [ ] Range of motion assessment
- [ ] Muscle strength assessment
- [ ] Plantar pressure analysis
- [ ] Treadmill and video gait analysis
- [ ] Computer based 2D technology (e.g. Gait scanner)
- [ ] Computer based 3D technology (e.g. Viacom)

Other (please specify)

Section 2: Intervention into flexible pes planus

This section is focused on determining when you would intervene in flexible pes planus in children during the course of your normal practice. You are asked to rate your 'likeliness' to intervene on the Likert scale below.

Whilst the preliminary survey indicated that panellists use alternative management strategies including: strengthening exercises, stretching, activity modification, footwear changes, strapping and anti-inflammatory medications etc. This survey is focused on the use of FOs for children. Please respond to the questions below considering that.

FOs, by definition, are in-shoe devices that influence the mechanics of the foot and lower limb. For the purpose of this survey, FOs may include pre-fabricated, accommodating or customised rigid or semi-rigid devices.

Please begin Section 2 below.

1. In the course of your normal practice, how likely are you to prescribe FOs for paediatric flexible pes planus in the presence of:

	Very Likely	Likely	Neutral	Unlikely	Very Unlikely
Moderate abnormal foot posture (i.e. 1 SD from expected measure)	○	○	○	○	○
Severe abnormal foot posture (i.e. 2 SDs from expected measure)	○	○	○	○	○
Reduced range of motion	○	○	○	○	○

Please provide some more information on the types of abnormal foot posture or reduced range of motion that may indicate a need for FOs.

2. In the course of your normal practice, you are how likely to intervention into paediatric flexible pes planus in the presence of:

	Very Likely	Likely	Neutral	Unlikely	Very Unlikely
Fatigue	◯	◯	◯	◯	◯
Perceived excessive tripping	◯	◯	◯	◯	◯
Clumsiness	◯	◯	◯	◯	◯
Diagnosed development coordination disorder	◯	◯	◯	◯	◯
Activity limitations	◯	◯	◯	◯	◯
Other foot function concerns (Please specify below)	◯	◯	◯	◯	◯

Please indicate any other functional concerns and provide some more information on your choices above of the foot function concerns that may indicate a need for FOs.

3. In the course of your normal practice, how likely are you to prescribe FOs for paediatric flexible pes planus in the presence of:

	Very Likely	Likely	Neutral	Unlikely	Very Unlikely
Foot pain	◯	◯	◯	◯	◯
Knee pain	◯	◯	◯	◯	◯
Back pain	◯	◯	◯	◯	◯
Lower limb pain	◯	◯	◯	◯	◯
Generalised lower limb pain	◯	◯	◯	◯	◯
Other reported pain (please specify in the comment box below)	◯	◯	◯	◯	◯

Please provide some more information on the presentations of pain that may indicate a need for FOs.

4. In the course of your normal practice, how likely are you to prescribe FOs for paediatric flexible pes planus in the presence of:

	Very Likely	Likely	Neutral	Unlikely	Very Unlikely
Parental concerns	○	○	○	○	○
Delayed milestones achievement	○	○	○	○	○
Family history of foot or lower limb disorders associated with flexible pes planus	○	○	○	○	○

Please provide some more information on your choices above e.g. parental concern and delayed milestones achievement that may indicate a need for intervention.

5. Please indicate if in course of your normal practice you perform any balance tests like hopping, jumping etc. to assess presence of pes planus in children.

6. Please comment on any other situation, if ever, you are likely to prescribe FOs for paediatric flexible pes planus in an otherwise normally developing child.

Section 3: Using foot orthoses for flexible pes planus in children

Well done, you are halfway!

This third section is focused on determining when and why, you would use FOs for children with flexible pes planus over other forms of intervention. FOs may be pre-fabricated or customised devices. Please answer the questions in light of your own clinical experience and your preferred choices clinically. Please remember that the focus is on paediatric clients only.

Please begin Section 3 below.

1. What particular age range do you consider appropriate to start using FOs for paediatric flexible pes planus?

- ◯ Age does not influence my decision
- ◯ 0-4 years
- ◯ 4-8 years
- ◯ 8-12 years
- ◯ 12-17 years

Please specify what guides your choice, if any, of that particular age.

2. Does the weight/mass of the child influence your decision to use FOs over other interventions? If so, what weight do you consider appropriate to starting using FOs for paediatric flexible pes planus?

- ○ Weight/mass does not influence my decision
- ○ > 10 kg
- ○ > 15 kg
- ○ > 20 kg
- ○ > 30 kg
- ○ 40+ kg

Please specify what guides your choice, if any, of that particular weight.

3. For treating paediatric flexible pes planus in otherwise normally developing children, are there any other considerations that would guide your decision to use FOs over other interventions?

4. Please indicate your desired outcomes from the use of FOs for paediatric flexible pes planus.

Section 4: Approach to prescription of foot orthoses used for flexible pes planus in children

This last section is focused on determining how FOs are prescribed when they are used in a paediatric flexible pes planus population. Please answer the questions in light of your own clinical experience and your preferred choices clinically.

Please begin section 4 below.

1. When you are prescribing FOs for otherwise normally developing children with flexible pes planus, what percentage of those FOs prescribed would be prefabricated devices?

0 50 100

2. When you are prescribing FOs for otherwise normally developing children with flexible pes planus, what percentage of those FOs prescribed would be customised devices?

0 50 100

3. When you are prescribing FOs for otherwise normally developing children with flexible pes planus, are there any other devices you would prescribe?

4. Please briefly summarise your preference, if any, for prefabricated or customised FOs for paediatric flexible pes planus?

If you only use prefabricated FOs for managing Paediatric Flexible Pes Planus and have selected 0% in the above question for Custom FOs then please continue to answer the questions on next page 'Prefabricated FOs for Paediatric Flexible Pes Planus'. You can then skip questions specific to customised FOs.

Conversely, if you have selected 0% in the above question for prefbricated FOs and only use custom FOs then please skip to page 9 'Customised FOs for Paediatric Flexible Pes Planus'.

Prefabricated Foot Orthoses (FOs) for Paediatric Flexible Pes Planus

The following questions are aimed to gain specific information on how prefabricated FOs are prescribed, please answer the questions in light of your own clinical experience and your preferred choices clinically.

1. Please indicate what features or characteristics guide your choice for prefabricated FOs specific for paediatric flexible pes planus and why?

2. Do you have any further comments to make on the use of prefabricated FOs for children with flexible pes planus?

Customised FOs for Paediatric Flexible Pes Planus

The following section relates to the prescription of customised FOs. From the preliminary survey the panel indicated that they prescribe the following 'types' of customised FOs for children with flexible pes planus: Modified Root style device, UCBL (University of California Biomechanics Laboratory) device and Blake (inverted) device.

For the purpose of this survey, we have defined these FOs by the following prescription variables:

- **Modified Root-style device (This device typically aims to hold the rearfoot in a vertical position, support the arch with minimal or standard expansion to this area and a forefoot post that aims to balance the forefoot perpendicular to the supporting surface)**
- **UCBL (University of California Biomechanics Laboratory)device. (This device typically has a higher heel cup and a medial and lateral flange than the modified Root-style device but still aims to hold the rearfoot in a vertical position, support the midfoot and 'balance' the forefoot as above)**
- **Blake (inverted) device (This device typically has a thickened medial expansion when compared to the modified Root-style device)**

The following questions aim to determine when, if ever, we use a 'standardised' prescription for children with flexible pes planus and when/why we modify the prescription for this population.

For the sake of consistency, we have used traditional manufacturing terminology throughout this survey. For example, traditional orthotic prescription forms give prescribers three choices of 'pour' based on the rearfoot bisection position when filling the negative cast i.e. inverted (including Blake devices), neutral/vertical or everted pour. With newer technology and computer scanning options the terminology varies however the concept remains that we correct to 'vertical' or 'invert/evert' from this point. Please see attached table in the email sent to you for terminology used. If you have any questions or queries regarding the terminology, please contact Sindhrani (0414 710 226) or Helen (0417 822 997) directly.

1. When prescribing customised FOs for paediatric flexible pes planus, please estimate how often you would prescribe the following individual prescription variables:

	0%	10%	20%	30%	40%	50%	60%	70%	80%	90%	100%
Neutral/vertical cast pour	○	○	○	○	○	○	○	○	○	○	○
Inverted cast pour (0-15 degrees)	○	○	○	○	○	○	○	○	○	○	○
Blake inverted device (≥ 15 degrees)	○	○	○	○	○	○	○	○	○	○	○
Everted cast pour	○	○	○	○	○	○	○	○	○	○	○

Please explain when (if ever) you would choose the **inverted** cast pour and why?

2. Please explain when (if ever) you would choose the**Blake inverted** cast pour and why?

3. When prescribing customised FOs for paediatric flexible pes planus, please estimate how often you would prescribe the following individual prescription variables:

	0%	10%	20%	30%	40%	50%	60%	70%	80%	90%	100%
Neutral/vertical rearfoot post	○	○	○	○	○	○	○	○	○	○	○
Inverted rearfoot post (0-15 degrees)	○	○	○	○	○	○	○	○	○	○	○
Blake inverted rearfoot post ($\geq$ 15 degrees)	○	○	○	○	○	○	○	○	○	○	○
Everted rearfoot post	○	○	○	○	○	○	○	○	○	○	○
Rearfoot post with motion	○	○	○	○	○	○	○	○	○	○	○

Please indicate when, if ever, you would prescribe an **inverted** rearfoot post?

4. Please indicate when, if ever, you would prescribe a **Blake inverted** rearfoot post?

5. Please indicate when, if ever, you would prescribe a **rearfoot post with motion**?

6. When prescribing customised FOs for paediatric flexible pes planus, please estimate how often you would prescribe the following individual prescription variable:

	0%	10%	20%	30%	40%	50%	60%	70%	80%	90%	100%
Medial heel (Kirby) skives (15 degrees)	○	○	○	○	○	○	○	○	○	○	○

Please explain when (if ever) you would use a medial heel skive and why?

7. When prescribing customised FOs for paediatric flexible pes planus, please estimate how often you would prescribe the following individual prescription variable:

	0%	10%	20%	30%	40%	50%	60%	70%	80%	90%	100%
UCBL (i.e. Medial and Lateral flange)	○	○	○	○	○	○	○	○	○	○	○

Please explain when (if ever) you would use this flange and why?

	0%	10%	20%	30%	40%	50%	60%	70%	80%	90%	100%
Medial flange only	○	○	○	○	○	○	○	○	○	○	○

Please explain when (if ever) you would use this flange and why?

	0%	10%	20%	30%	40%	50%	60%	70%	80%	90%	100%
Lateral flange only	○	○	○	○	○	○	○	○	○	○	○

Please explain when (if ever) you would use this flange and why?

8. Please indicate what best represents your choice of medial plaster expansion (a.k.a arch fill) for for paediatric flexible pes planus?

	0%	10%	20%	30%	40%	50%	60%	70%	80%	90%	100%
Minimal arch fill	◯	◯	◯	◯	◯	◯	◯	◯	◯	◯	◯

Please specify when (if ever) you would use this arch fill and why?

	0%	10%	20%	30%	40%	50%	60%	70%	80%	90%	100%
Standard arch fill	◯	◯	◯	◯	◯	◯	◯	◯	◯	◯	◯

Please specify when (if ever) you would use this arch fill and why?

	0%	10%	20%	30%	40%	50%	60%	70%	80%	90%	100%
Maximum arch fill	◯	◯	◯	◯	◯	◯	◯	◯	◯	◯	◯

Please specify when (if ever) you would use this arch fill and why?

9. Please indicate what best represents your choice of forefoot posting for paediatric flexible pes planus?

	0%	10%	20%	30%	40%	50%	60%	70%	80%	90%	100%
No forefoot post	◯	◯	◯	◯	◯	◯	◯	◯	◯	◯	◯
Forefoot balanced to perpendicular	◯	◯	◯	◯	◯	◯	◯	◯	◯	◯	◯
Inverted forefoot post	◯	◯	◯	◯	◯	◯	◯	◯	◯	◯	◯
Everted forefoot post	◯	◯	◯	◯	◯	◯	◯	◯	◯	◯	◯

Please indicate the characteristics of forefoot post if no post, or an inverted or everted forefoot post is used.

10. Please estimate below the percentage of devices you prescribe using the following shell materials for paediatric flexible pes planus.

	0%	10%	20%	30%	40%	50%	60%	70%	80%	90%	100%
Polyolyenes (e.g. polypropylene)	◯	◯	◯	◯	◯	◯	◯	◯	◯	◯	◯
Cellular foam (e.g. EVA)	◯	◯	◯	◯	◯	◯	◯	◯	◯	◯	◯
Composite (e.g. carbon graphite)	◯	◯	◯	◯	◯	◯	◯	◯	◯	◯	◯
Other (please specify) with the percentage estimate	◯	◯	◯	◯	◯	◯	◯	◯	◯	◯	◯

Other (please specify) with the percentage estimate

11. Please indicate any further modifications or prescription variables you routinely use when prescribing customised FOs specifically for the paediatric flexible pes planus population?

Thank you

Thank you for taking time to complete this round 1 of the Delphi survey. Your time and participation is really appreciated.

Please email Sindhrani Dars at <darsy009@mymail.unisa.edu.au> if you have any queries. Also, please note that the following rounds will be less time consuming and will be sent in the same format as this round.

Thank you again.

1. Please add your name below in the comment box. Your participation will still remain anonymous. The reason for requiring name here is just to enable us to send Delphi Round 2 to you.

2. If you would like to receive your responses for this round via an email then please provide your email address.
