# Supplementary material for: When, why and how foot orthoses (FOs) should be prescribed for children with flexible pes planus: a Delphi survey of podiatrists
Source: PeerJ. 2018 Apr 16;6:e4667. doi: 10.7717/peerj.4667 (PMC5907774; doi:10.7717/peerj.4667)
Supplement: Data S3 [file peerj-06-4667-s003.pdf]

University of South Australia

A Delphi consensus of Podiatrists for prescription of Foot Orthoses (FOs) for children with flexible pes planus

Additional file 4: Glossary of terms (Prescription variables)

Prescription variable	Method of manufacture
Cast Pour	Negative cast is held in the prescribed position, based on a bisection of the posterior heel relative to the supporting surface, while liquefied plaster is poured into negative cast
Inverted	Indicates that the negative cast, when poured, is held in an inverted position relative to the heel bisection
Neutral	Indicates that the negative cast, when poured, is held in a vertical position relative to the heel bisection
Everted	Indicates that the negative cast, when poured, is held in an everted position relative to the heel bisection
Medial heel (Kirby) skive	Indicates a small amount of plaster is skived away from the medial heel of the positive cast (skive is generally angled 15 degrees varus/inverted to the plane of the plantar surface of the forefoot post)
Rearfoot post	An addition, typically fashioned from a heat mouldable material, that is applied to the final orthosis to stabilise the heel in a vertical position or angle it in the frontal plane (also known as a heel stabiliser)
No post	No external rearfoot post
Extrinsic	An external heel post that stabilises the orthosis in a vertical position
Extrinsic (inverted)	An external heel post that tilts the orthosis into an inverted position
Extrinsic (everted)	An external heel post that tilts the orthoses into an everted position
Extrinsic (with motion)	An external heel post that has a bi-planar grind on the plantar aspect
Arch fill	The plaster expansion applied to the medial longitudinal arch area of the positive cast
Minimal	A decreased plaster expansion
Standard	A standard plaster expansion
Maximum	An increased plaster expansion
Flange	A midfoot extension of the final orthosis border, typically prescribed in conjunction with a deep and distally extended heel cup
Medial	A superomedial extension
Lateral	A superolateral extension
Forefoot post	A corrective reference platform applied to the medial and/or lateral forefoot
Balanced to perpendicular	The reference platform applied to the plantar forefoot to hold the forefoot alignment as parallel to the supporting surface and perpendicular to the rearfoot
Intrinsic	The reference platform is applied to the positive cast
Extrinsic	The platform is applied to the shell of the final orthosis
No post	No reference platform applied

Notes: MTPJ = metatarsophalangeal joint. Negative cast - plaster cast impression of the foot (generally made from plaster or paris bandage), Positive cast - plaster mould of the foot that is formed as the result of liquefied plaster poured into the negative cast.
