# Supplementary material for: When, why and how foot orthoses (FOs) should be prescribed for children with flexible pes planus: a Delphi survey of podiatrists
Source: PeerJ. 2018 Apr 16;6:e4667. doi: 10.7717/peerj.4667 (PMC5907774; doi:10.7717/peerj.4667)
Supplement: Data S4 [file peerj-06-4667-s004.pdf]

Additional file 5 – All excluded statements

Excluded statements from all three rounds of Delphi survey of Podiatrists on paediatric flexible pes planus

Excluded statements from Round 1

Variable	Options	Level of consensus
Assessment outcomes routinely used to determine the presence of a flexible pes planus foot posture;	Foot print indices (e.g. arch height index, Staheli's arch index)	6.7%
	Diagnostic imaging (e.g. x-rays, CT scans, MRI)	6.7%
Static foot posture measures routinely used to determine the presence of a flexible pes planus foot posture in children;	I do not determine static foot posture	15.4%
	Navicular height (truncated to foot length)	30.8%
	Navicular drift	38.5%
Static Foot Posture measure is by;	I do not determine static foot posture	13.3%
	Tractograph	46.7%
	Gravity goniometer	13.3%
Techniques routinely used to determine foot function in paediatric flexible pes planus populations;	I do not determine foot function	0.0%
	Supination resistance	46.7%
	Plantar pressure analysis	33.3%
	Treadmill and video gait analysis	33.3%
	Computer based 2D technology (e.g. Gait scanner)	6.7%
	Computer based 3D technology (e.g. Viacom)	6.7%
How likely are FOs prescribed for paediatric flexible pes planus in the presence of;	Moderate abnormal foot posture (i.e. 1 SD from expected measure)	66.6%
	Reduced range of motion	35.7%
How likely is intervention into paediatric flexible pes planus in the presence of:	Clumsiness	46.6%
	Back pain	42.9%
	Parental concerns	0.0%

	Delayed milestones achievement	26.6%
	Family history of foot or lower limb disorders associated with flexible pes planus	33.3%
Age range to start FOs prescription for paediatric flexible pes planus	0-4 years	6.7%
	4-8 years	33.3%
	8-12 years	6.7%
	12-17 years	0.0%
Does the weight/mass of the child influence your decision to use FOs over other interventions?	> 10 kg	0.0%
	> 15 kg	7.7%
	> 20 kg	0.0%
	> 30 kg	0.0%
	40+ kg	0.0%
Percentage of customised devices	Average = 31% (Range 0-100%)	31.0%
Individual prescription variables of custom FOs	Inverted cast pour (0-15 degrees), No consensus on use of more than 70%	53.9% (0% use)
	Neutral/vertical rearfoot post, No consensus on use of more than 70%	30.8% (0% use)
	Inverted rearfoot post (0-15 degrees), No consensus on use of more than 70%	58.3% (0% use)
	Medial heel (Kirby) skive – 15 degrees, No consensus on use of more than 70%	46.2% (0% use)
	UCBL (i.e. Medial and Lateral flange), No consensus on use of more than 70%	38.5% (0% use)
	Medial flange only, No consensus on use of more than 70%	38.4% (0% use)
	Lateral flange only, No consensus on use of more than 70%	55.6% (0% use)
Choice of medial plaster expansion (a.k.a arch fill) for paediatric flexible pes planus?	Standard arch fill (≥70% likely to use it)	25.0%
Forefoot posting for paediatric flexible pes planus;	No forefoot post	62.5% (0% use)
Shell materials for paediatric flexible pes planus.	Polyolyenes (e.g. polypropylene)	41.7% (≥70% likely to use)
	Composite (e.g. carbon graphite)	54.5% (0% use)

Excluded statements from Round 2

**Variable**	**Options**	**Agreement**

The presence of a flexible pes planus foot posture can be determined by:	Review of proximal components (e.g. hip position, femoral torsion etc.) to assess for compensation of foot pronation	35.7%
The following static foot posture measures are appropriate to determine paediatric flexible flat foot:	Lateral foot concavity	28.6%
The balance tests performed to assess function with paediatric flexible pes planus should include;	BOT-2 assessment	28.6%
The likeliness of prescribing FOs for paediatric flexible pes planus increases:	With a family history of lower limb problems due to pes planus	35.7%
The likeliness of intervening in paediatric flexible pes planus increases with:	Growing pains	35.7%
	Night cramps	35.7%
To the prescription of FOs for paediatric flexible pes planus in an otherwise normally developing child, is likely if:	There is trauma from footwear like blistering	38.5%
	Shoes are being damaged rapidly	42.9%
In relation to the age of the child, the decision to prescribe FOs for paediatric flexible pes planus is:	Appropriate when a child is able to self-report pain	38.5%
	Appropriate if child's age is greater than 14 months of age	15.4%
In relation to the weight/mass of the child, the decision to prescribe FOs paediatric flexible pes planus is influenced by:	The child’s BMI, with larger children FOs prescribed earlier	30.7%
For treating paediatric flexible pes planus in otherwise normally developing children, considerations that would guide decision to use FOs over other interventions, include:	Family history of painful pes planus	23.1%
	Parents understanding of the problem and willingness to start FOs.	30.8%
	Unwillingness of child/family in first line physical therapy and exercise	38.5%
	Socio-economic reasons	23.1%
	Sports or activity engagement demands.	46.2%
The aim of prescribing FOs for paediatric flexible pes planus is to:	Gain compliance with desire/choice of child to wear orthoses and Footwear	30.8%
	Improve plantar pressure	38.5%
	Maintain a vertical calcaneus	7.7%
	Gain parental satisfaction	7.7%
When comparing pre-fabricated FOs to custom-made FOs, it is considered that	Pre-fabricated FOs offer lower weight, better fit, more design and material choices/sizes	23.1%
	Pre-fabricated FOs are more predictable	23.1%
	Customised FOs are to be used for high weight children and/or increased activity requirement	23.1%

	Customised FOs might work better to stabilise the rearfoot including Talonavicular joint and encourage the plantarflexion of the midfoot by encouraging lateral compartment muscles	15.4%
The features or characteristics that guide the choice of prefabricated FOs specific for paediatric flexible pes planus may include:	Flexibility	30.8%
	Medial flange	23.1%
	Decent arch fill	46.2%
	Heel skive	15.4%
	Cuboid notch	15.4%
With regards to the use of prefabricated FOs for children with flexible pes planus:	Pre-fabricated FOs since cheaper are good to identify compliance before moving to expensive Custom FOs	46.2%
	Pre-fabricated soft orthoses and old distorted orthoses lead to forefoot varus alignments and a dependence of treatment	8.3%
When prescribing Custom FOs for paediatric flexible pes planus, an inverted cast pour may be used if there is:	Excessive Rearfoot ROM in Frontal plane	41.7%
	Lateral ankle impingement	41.7%
	Midfoot Abduction	33.3%
	A need to hold STJ close to neutral	33.3%
When prescribing Custom FOs for paediatric flexible pes planus, a Blake's Inverted cast pour may be used in presence of:	Excessive pes planus	30.8%
When prescribing Custom FOs for paediatric flexible pes planus, an inverted rearfoot post may be used if:	Depending on available joint range of motion, dynamic foot motion in gait and particular activity demands	28.3%
	In the presence of TNJ instability	25.0%
	To stabilise the rearfoot	41.7%
When prescribing Custom FOs for paediatric flexible pes planus, a rearfoot post with motion may be used:	To achieve control at sustentaculum tali and motion quality through first and second rockers	28.3%
When prescribing Custom FOs for paediatric flexible pes planus, a Medial (Kirby) heel skive may be used:	To reduce pressure on midfoot making orthoses smaller and easier to fit in footwear	33.3%
When prescribing Custom FOs for paediatric flexible pes planus, a UCBL (i.e. Medial and Lateral flange) device may be used:	In very flexible pes planus where medial edge of device is not tolerated	58.3%
	When there is instability in gait and increased incidence of lateral ankle sprain	38.5%
	Developmental Coordination Disorder	61.5%
	For better tolerance	38.5%

When prescribing Custom FOs for paediatric flexible pes planus, a medial flange device may be used:	When there is high navicular drift	38.5%
When prescribing Custom FOs for paediatric flexible pes planus, a lateral flange device may be used:	In severe MF break	36.4%
	To prevent lateral slippage of the orthoses	33.3%
When prescribing Custom FOs for paediatric flexible pes planus, a minimal arch fill may be used:	To allow for skin expansion	33.3%
When prescribing Custom FOs for paediatric flexible pes planus, a standard arch fill may be used:	In extreme pes planus where more stability is required than control	33.3%
	To get the arch height similar to NCSP	25.0%
	For less severe pes planus	33.3%
When prescribing Custom FOs for paediatric flexible pes planus, a maximum arch fill may be used:	Where there is more fat pad i.e. overweight child	41.7%
	To get comfortable midfoot height to avoid blistering/irritation	8.3%
	Where skin trauma, caused by orthoses, has previously occurred	3.8%
When prescribing, forefoot posting for Custom FOs for paediatric flexible pes planus; the following should be considered:	Natural transverse arch should be captured to provide intrinsic metatarsal dome of the balanced device	33.3%
	FF posting usually used to correct rearfoot position	33.3%
	If there is a plantarflexed 1st ray, forefoot post should be lateral to make the forefoot abducted (locked calcaneo-cuboid joint) and rearfoot inverted	25.0%
When choosing shell materials for Custom FOs for paediatric pes planus, the following could be considered;	EVA for improved comfort	46.2%
For flexible pes planus in otherwise normally developing children, alternative devices to FOs may include;	Triplanar wedges	46.2%
	Footwear modification	46.2%
When prescribing custom FOs for paediatric flexible pes planus, the following should be considered;	At times, a first ray cut-out	33.3%
	Valgus filler pads to cushion arch area allowing blister prevention	9.1%
	5th ray cut out to allow a slimmer fit in footwear	9.1%

Excluded statements from Round 3

Variable	Options	Agreement
The following static foot posture measures are appropriate to determine paediatric flexible flat foot:	Other static components of foot not covered in FPI (e.g. arch rise, calcaneal inclination etc.)	57.1%

	Comparison of one foot to another	53.8%
The following techniques are appropriate to determine foot function in paediatric flexible pes planus:	Jack's Test	50.0%
The balance tests performed to assess function with paediatric flexible pes planus should include;	Squatting	64.3%
	Skipping	42.9%
	Reflexes	50.0%
The prescription of FOs for paediatric flexible pes planus in an otherwise normally developing child, is likely if:	There is trauma from footwear like blistering	50.0%
The likeliness of prescribing FOs for paediatric flexible pes planus increases:	Parental concerns (e.g. inability to keep up with peers and lack of confidence for participation)	57.1%
The aim of prescribing FOs for paediatric flexible pes planus is to:	Reduce trauma to structures at risk such as spring ligament	53.8%
	Foot posture retraining and improvement	42.9%
When comparing pre-fabricated FOs to custom-made FOs, it is considered that	There is no evidence that one is more effective than the other	42.9%
	Pre-fabricated FOs should be used when they offer enough short term symptomatic relief in acute presentations for no longer than 6/12	61.5%
The features or characteristics that guide the choice of prefabricated FOs specific for paediatric flexible pes planus may include:	Deep heel cup	57.1%
	Gecko type device for gross pronation	14.3%
When prescribing Custom FOs for paediatric flexible pes planus, an inverted cast pour may be used if there is:	Additional control required	53.9%
	Gross talar pronation	23.1%
When prescribing Custom FOs for paediatric flexible pes planus, an inverted rearfoot post may be used if:	To reduce STJ pronation	64.3%
When prescribing Custom FOs for paediatric flexible pes planus, a UCBL (i.e. Medial and Lateral flange) device may be used:	In very flexible pes planus where medial edge of device is not tolerated	50.0%
	Developmental Coordination Disorder	57.1%
When prescribing Custom FOs for paediatric flexible pes planus, a medial flange device may be used:	To accommodate midfoot break, wider than vertical to prevent blistering/irritation on TNJ region	61.5%
When prescribing Custom FOs for paediatric flexible pes planus, a lateral flange device may be used:	To reduce risk of lateral ankle sprains or functional lateral ankle Instability	50.0%
When prescribing Custom FOs for paediatric flexible pes planus, a minimal arch fill may be used:	Where significant navicular drop with flattened MLA exists, i.e. severe paediatric flexible pes planus	64.3%
	For better tolerance	64.3%

When prescribing Custom FOs for paediatric flexible pes planus, a standard arch fill may be used:	To allow for skin/fat pad expansion	64.3%
For flexible pes planus in otherwise normally developing children, alternative devices to FOs may include;	Padded insoles/in-shoe padding	50.0%
